# Strategies of NaCl Tolerance in Saline–Alkali-Tolerant Green Microalga *Monoraphidium dybowskii* LB50

**DOI:** 10.3390/plants12193495

**Published:** 2023-10-07

**Authors:** Haijian Yang, Jing Zhang, Hua Li

**Affiliations:** 1 Key Laboratory of Algal Biology, Institute of Hydrobiology, Chinese Academy of Sciences, Wuhan 430072, China; hjyang@ihb.ac.cn; 2 Analysis and Testing Center of Institute of Hydrobiology, Chinese Academy of Sciences, Wuhan 430072, China

**Keywords:** NaCl tolerance, *Monoraphidium dybowskii* LB50, triacylglycerol, proteomic, carbon partitioning

## Abstract

Studying how freshwater cells modify metabolism and membrane lipids in response to salt stress is important for understanding how freshwater organisms adapt to salt stress and investigating new osmoregulatory ways. Physiological, biochemical, metabolic, and proteomic analyses were applied in a novel saline–alkali-tolerant microalga *Monoraphidium dybowskii* LB50 under different NaCl concentrations. Cells adopt a variety of strategies to adapt to salt stress, including increasing ion transport and osmolytes, regulating cell cycle and life history, and accumulating triacylglycerol (TAG). A large number of metabolic activities point to TAG accumulation. With increasing NaCl concentration, the C resource for TAG accumulation went from photosynthetically fixed C and a small amount of lipid remodeling to macromolecule degradation and a mass of lipid remodeling, respectively. The energy for TAG accumulation went from linear electron transfer and oxidative phosphate pentose pathway to cyclic electron flow, substrate phosphorylation, oxidation phosphorylation, and FA oxidation. Additionally, digalacturonic acid and amino acids of the N-acetyl group, which usually were the osmotica for marine organisms, were important for *M. dybowskii* LB50. Freshwater organisms evolved many biological ways to adapt to salt stress. This insight enriches our understanding of the adaptation mechanisms underlying abiotic stress.

## 1. Introduction

The evolution of life on early Earth occurred in extremely high salt aqueous environments such as hydrothermal vents [1], acid-brine oceans, or brine surfaces [2,3]. Hypersaline brines have also remained a stable habitat throughout the planet’s geological history [4]. Even hypersaline environments with salt concentrations up to NaCl saturation are inhabited by halophilic and highly halotolerant representatives of all three domains of life: archaea, bacteria, and eukarya [5]. Therefore, NaCl-saturated brines are thermodynamically moderate, rather than extreme, for life [6]. Factors like cycles of flooding and the evapoconcentration of soil salts caused by seasonal changes in temperature and sun irradiance also result in dynamic fluctuations in environmental salt concentrations perceived by plants. Recent research has identified that all plants can tolerate NaCl in the medium in which they grow, varying considerably from under 2.9 to 58.5 g L^−1^ [7]. Various freshwater organisms also have salt tolerance during evolution [8]. Therefore, understanding the basic mechanisms underlying cellular response and the adaptation of freshwater organisms under salt stress has significance for studying the origin and evolution of organisms and will enable the rational utilization of saline–alkali water resources for algae cultivation.

Osmotic and ionic stresses, as well as secondary stress caused by the first two stresses, are three NaCl-induced stress pathways [9]. To cope with the NaCl environmental stress, cells have evolved multiple adaptive strategies, such as accumulation osmolytes [5,9]; excluding harmful ions; reducing the intake of salt ions via the changes in cell membrane characteristics and other transport systems [10]; scavenging reactive oxygen species; preventing oxidative cellular damage via triacylglycerol (TAG) metabolic and antioxidant synthesis [11]; and storing excessive energy by accumulating lipid and TAG [12]. Therefore, lipid, membrane lipid, and TAG play important roles under NaCl stress. Meanwhile, many microalgae form and accumulate lipids in the form of TAG by altering the lipid biosynthesis pathway under salt or NaCl stress [13,14,15]. TAG accumulation under suboptimal conditions may play biological roles, such as providing a fitness advantage for the cells under suboptimal nutrient conditions. Recently, studies have shown that lipid metabolism mediates salt stress tolerance [16,17].

Osmotic stress is the most important of these stresses. Osmotic substances have always been the focus of research, from archaea to plants. Different strategies exist to provide the osmotic balance of the cells’ cytoplasm with the salinity of the medium. One option used by many halophilic archaea (*Halobacteria* requires at least 150–200 g L^−1^ salts) and a few representatives of the bacteria is to accumulate salts, mainly KCl, and adapt the entire intracellular machinery in functioning in the presence of the molar concentrations of salts [18]. A considerably widespread option is the synthesis or accumulation of organic osmotics, which are the so-called compatible solutes. Marine strain *Halobacillus halophilus* that can grow at between 29.25 and 117 g L^−1^ NaCl uses inorganic ions and organic osmotic solutes for osmotic adaptation, including Cl^−1^, glutamine and glutamate, proline and ectoine, N-acetyl ornithine, and N-acetyl lysine [19]. Similar to most groups of the bacteria, cyanobacteria use organic osmotic solutes, such as glucosylglycerol, glycine betaine (halophilic and marine cyanobacteria), sucrose, and trehalose (halotolerant and euryhaline organism strains) to adjust the cytoplasm with the external medium osmotically [20,21]. Only several halophilic and halotolerant eukaryotic microorganisms, such as algae, such as *Dunaliella* and *Asteromonas* (from salt lakes or salt marshes that grows on salt concentrations from 29.25 to 321.75 g L^−1^), and many fungi and yeasts used glycerol as an osmotic solute [22]. Plants, such as halophytes and terrestrial plants, accumulated several classes of compatible osmolytes under salt stress as follows: charged metabolites, including proline, glycine betaine, β-alanine betaine, choline-O-sulfate, hydroxyproline, dimethylsulfonium propionate, and putrescine; polyols, including mannitol, myoinositol, ononitol, pinitol, and sorbitol; and sugars, including sucrose, fructose, trehalose, raffinose, and fructans [7,9]. The type of compatible osmolytes synthesized under salt stress is at least partly species-specific, and these metabolites may also directly bind to and activate/inactivate enzymes to regulate salt response. However, although a considerable amount of research has been conducted on the adaptation mechanisms of holophilic and salt-tolerant plants and microorganisms, the adaptive strategies to high salinity in freshwater unicellular plants are still largely unknown.

The freshwater green algae are lower plants that are important members of the process of aquatic evolution to terrestrial plants [23]. Several green algae are highly tolerant of osmosis [5]. *Monoraphidium dybowskii* LB50 is a saline–alkali-tolerant and oil-rich freshwater green alga [8,24]. Therefore, in the present study, the unicellular freshwater green alga *M. dybowskii* LB50 was used as a material to reveal the whole-cell adaptation strategies along a salinity gradient. We focused on the various changes under different NaCl stress concentrations. The changes in cell morphology and lipid droplets under different NaCl concentrations were observed via microscopy and ultramicroscopy. Intermediate metabolites, enzymes, and differentially expressed proteins (DEPs) were analyzed via physiological, biochemical, metabolomic, and proteomic analyses. This research provides additional insights into the responses of freshwater organisms to NaCl stress, assisting in the rational genetic engineering of halotolerant microorganisms and showing how salinity drives plants to adapt in increasingly salinized environments.

## 2. Results

### 2.1. Physiological Shifts to NaCl Concentrations

Our previous results indicated that *M. dybowskii* LB50, which can tolerate salinity from 0 to 40 g L^−1^ NaCl concentrations, is the oleaginous freshwater microalga [8]. The TAG or lipid production of *M. dybowskii* LB50 is enhanced by 20–60 g L^−1^ NaCl induction. Lipid production in other microalgae is also enhanced by different NaCl concentrations. To explore the adaptation mechanism of this strain along a salinity gradient, we added different amounts of NaCl to prepare 0 g L^−1^ (control group, CG), 20 g L^−1^ (low NaCl concentration, L), 40 g L^−1^ (medium NaCl concentration, M), and 60 g L^−1^ (high NaCl concentration, H) solutions for the culture.

**Figure 1:**
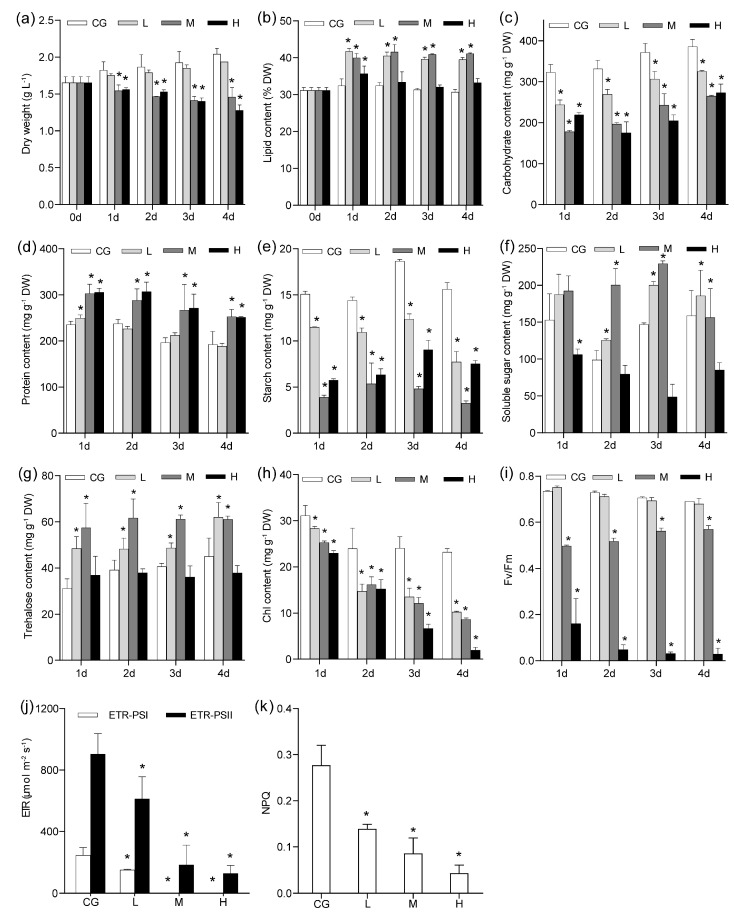
Influence of different NaCl concentrations on biomass productivity (**a**), lipid content (**b**), carbohydrate content (**c**), protein content (**d**), starch content (**e**), soluble sugar content (**f**), trehalose content (**g**), chlorophyll content (**h**), and Fv/Fm (**i**). ETR (**j**) and NPQ (**k**) in *Monoraphidium dybowskii* LB50 after 1 day under different NaCl concentrations. Values are the means and SD of three biological replicates. CG, 0 g L^−1^ NaCl concentration; L, 20 g L^−1^ NaCl concentration; M, 40 g L^−1^ NaCl concentration, H, 60 g L^−1^ NaCl concentration. ETR, electron transport rate; NPQ, non-photochemical quenching. Asterisks indicate statistically significant differences compared with control conditions (*p* < 0.05).

Growth was significantly reduced (*p* < 0.05) under M and H conditions, whereas growth did not significantly decrease under L conditions. With regard to lipids, the highest lipid content (~40%) was observed under L and M conditions. Lipid content was also significantly higher under H conditions than in the control but lower than under other conditions (Figure 1). Carbohydrate contents (including starch) were significantly lower under NaCl concentrations than those in the control (Figure 1). These findings suggested that a portion of carbohydrates was distributed in accumulated lipids during the C flow. Protein content was not significantly affected under L conditions and was higher under M and H conditions than in the control. 

A certain number of osmotic regulation substances significantly changed (Figure 1f–h). The amounts of soluble sugar and trehalose increased under L and M conditions but decreased under H conditions. Chlorophyll and variable fluorescence (Fv)/maximal fluorescence (Fm) did not significantly change after 1 day under L conditions, whereas they significantly decreased under M and H conditions (*p* < 0.05). This finding indicated that the chloroplast was not affected under L conditions and collapsed under M and H conditions (Figure S1). Transmission electron microscopy (TEM) results showed that lipid droplets were the most abundant after 1 day under L conditions (Figure S1), and the chloroplast gradually disintegrated with increasing NaCl concentrations. The cell wall (CW) became rough and thick. The time points after 1 day of NaCl concentrations were chosen for further experiments. Meanwhile, the electron transport rate (ETR, in photosystems I and II (PSI and PSII)) decreased gradually. Under M and H treatments, the ETR of PSII was 0 (Figure 1), suggesting that the cells relied heavily on cyclic electron flow for ATP production.

### 2.2. Lipid Ratios and Fatty Acid (FA) of Microalgae to NaCl Concentrations

**Figure 2:**
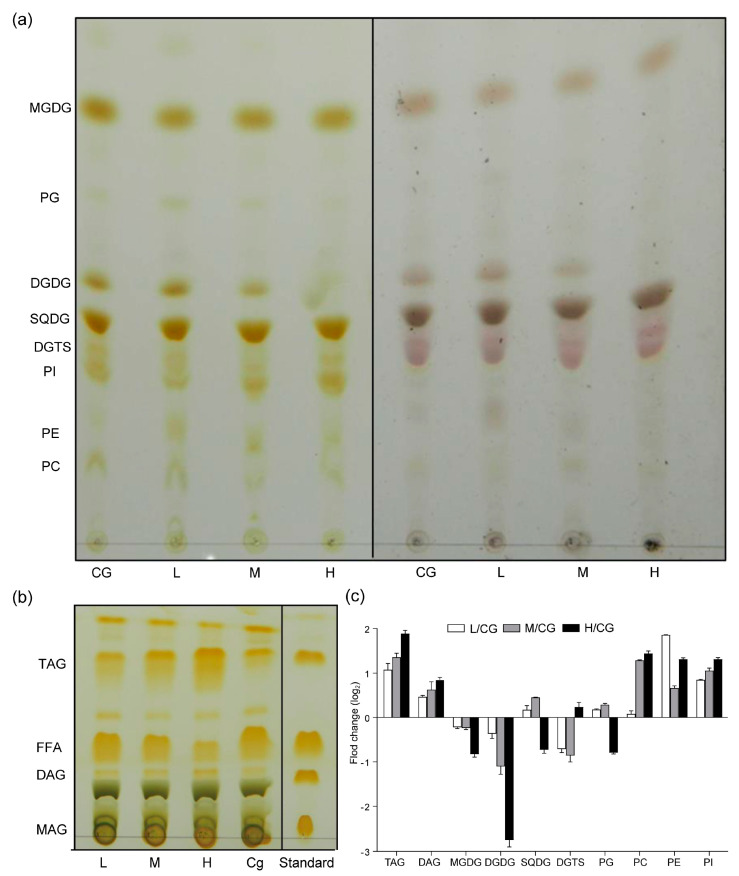
Alterations in polar (**a**) and neutral lipids (**b**) and fold change in treatments relative to the control in lipid composition (**c**) after 1 day under different NaCl concentrations. CG, 0 g L^−1^ NaCl concentration; L, 20 g L^−1^ NaCl concentration; M, 40 g L^−1^ NaCl concentration, H, 60 g L^−1^ NaCl concentration.

**Figure 3:**
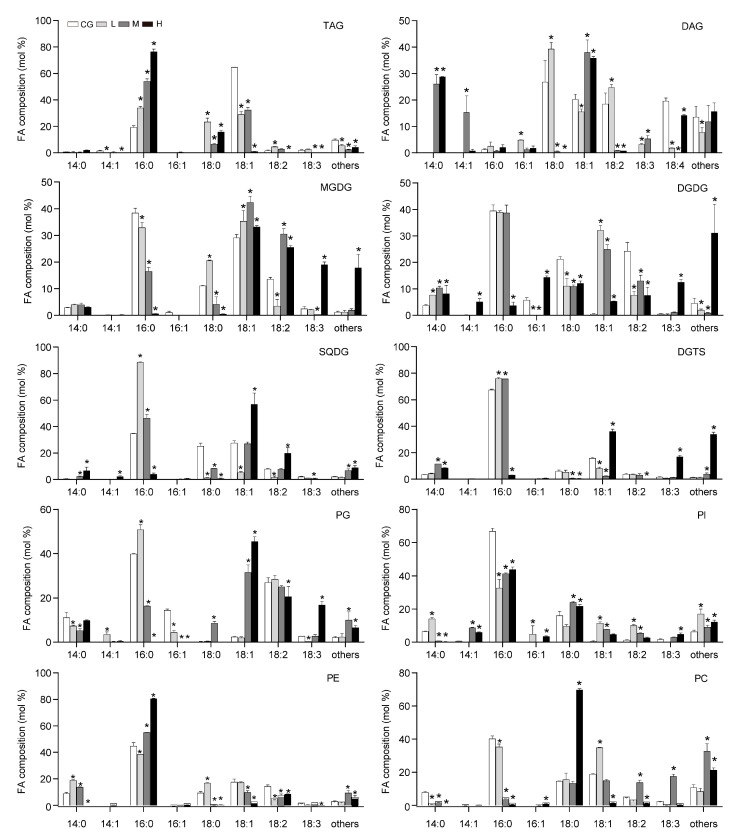
Fatty acid composition of lipid classes in *Monoraphidium dybowskii* LB50. White bars, CG (0 g L^−1^ NaCl concentration); light grey bars, L (20 g L^−1^ NaCl concentration); dark gray bars, M (40 g L^−1^ NaCl concentration); and black bars, H (60 g L^−1^ NaCl concentration). Values are the means and SD of three biological replicates. Asterisks indicate statistically significant differences compared with the control conditions (*p* < 0.05).

According to the results in Figure 1, the changes in the lipid were extremely significant, and the cell membrane may change the mobility of the cell membrane by changing FAs to adapt to the NaCl environment. Neutral lipids were separated into monoacylglycerols, DAG, TAG, and free FAs (FFAs). The TAG content (% lipid) significantly increased (Figure 2), whereas the FFA content (% lipid) decreased. The polar lipids were separated well into glycolipids (MGDG, DGDG, and SQDG), phospholipids (PG, PI, PE, and PC), and diacylglyceryltrimethylhomoserine (DGTS) and showed that the DGDG content (% lipid) decreased, whereas the PE increased, which was consistent with the result of Yang et al. [8]. With increasing NaCl concentrations, the FAs of the lipid fractions were mainly C16:0 and C18:1. The C16:0 fraction of TAG increased, whereas the C18:1 and polyunsaturated FA contents decreased. Figure 3 shows that the long-chain FAs (saturated FA, SFA) of glycolipids and phospholipids significantly increased under M and H conditions, suggesting that the cell membrane synthesized long C chains to protect the cells.

The SFA of the total lipid and TAG increased, but monounsaturated and polyunsaturated FAs decreased. Therefore, the degree of unsaturation (DU) decreased (Table 1). The DU of the DGDG decreased first and then increased, and its content decreased with NaCl concentration. This result was the opposite of the DU of PC. The membrane was stimulated to increase the fluidity and permeability at an appropriate salt concentration, whereas fluidity and permeability decreased to protect the cells at high concentrations.

### 2.3. Osmolytes of M. dybowskii LB50 to NaCl Concentrations

Metabolic profiling techniques appear to be compatible with studies aimed at elucidating the mechanisms underlying metabolic changes in microalgae exposed to various environmental stimuli, such as salt stress. In this study, intracellular metabolites were extracted and analyzed using gas chromatography–mass spectrometry (GC/MS), and variations in metabolite amounts were monitored as described previously. The detectable peaks of the samples were 1033 in total, and the internal standard was used for data quality control (reproducibility). The detectable metabolites of *M. dybowskii* LB50 in GC/MS were 301 in total, 169 of which were differential metabolites (Data S1).

**Figure 4:**
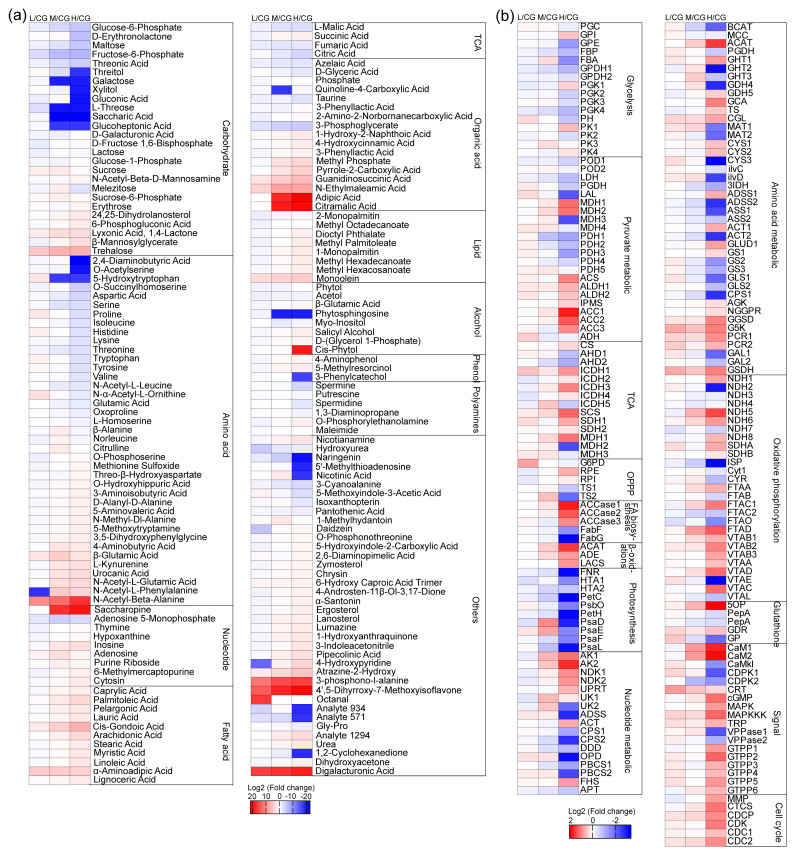
Heatmaps of differential metabolites (**a**) and differentially expressed proteins (**b**) under different NaCl concentrations. CG, 0 g L^−1^ NaCl concentration; L, 20 g L^−1^ NaCl concentration; M, 40 g L^−1^ NaCl concentration, H, 60 g L^−1^ NaCl concentration. Each value was divided by the corresponding control and log_2_-transformed values. The full names of the corresponding enzymes are provided in Data S1.

In general, carbohydrates and amino acids decreased, and lipids increased. The changes in metabolites under L conditions did not significantly change (Figure 4a). Under L conditions, most of the FAs decreased slightly, but palmitoleic acid, arachidonic acid, and cis-gondoic acid increased. Among these acids, cis-gondoic acid showed the most significant increase. Under M and H conditions, most of the FAs increased. The increase in palmitoleic acid and linoleic acid was the most evident, and this finding was consistent with the results of the FA analysis. The change in FAME was similar to FAs.

In response to salt stress, glycerol and proline, which are the osmotic regulators in Daniella, did not show the most remarkable change. Galacturonic acid, trehalose, N-acetyl-β-alanine, and organic acids, especially galacturonic acid and N-acetyl-β-alanine, strongly increased with increasing salt concentration. Galacturonan, which is soluble in water due to its multiple hydroxyl groups and carboxylic acids, can be degraded by the CW of the green algae and then transported into the cell to increase the osmotic potential in the cytoplasm. The degradation of galacturonic acid into galactose was not observed. Galacturonic acid and N-acetyl-β-alanine were possibly the osmotic regulators used in order to cope with salt stress.

### 2.4. Metabolic Response to NaCl Concentrations by Integrating Proteomics and Metabolic Profiling Data

#### 2.4.1. Quantitative Proteomic

To analyze the mechanism underlying the metabolite accumulation of *M. dybowskii* LB50 under NaCl stress further, we identified the differential proteins after 1 day of NaCl stress. A total of 1321 proteins were identified (Data S2). The number of DEPs increased with increasing NaCl concentration (Data S3). The proportion of the downregulated proteins also increased (Table S1). 

To determine the functional distribution of such proteins, we performed a functional annotation (GO term). The main biological processes (Figure S2) were metabolic and cellular processes, followed by a response to a stimulus. The upregulated proteins increased and the downregulated proteins decreased with the increasing NaCl concentrations. In terms of molecular function, catalytic activity and binding, which were directly related to salt-induced osmotic responses, occupied the main proportion. The change law of the up- or downregulated proteins was similar to the biological process. The number of DEPs in the growth process was also relatively small. Combined with the annotation of the cluster of orthologous groups of proteins (COGs), the proportion of the 19 other categories of function increased with increasing salt concentration, except for chromatin structure and dynamics (Figure S2). The proportion of lipid metabolism and transport was relatively low. The results of the GO terms showed that the cells were mainly responsive to salt stress and metabolism.

#### 2.4.2. Signal Response to NaCl Concentrations

To mount an effective response to cope with salt stress, the cells developed the ability to sense the hyper-osmotic component and ionic Na^+^ component of the stress. Zhao et al. [25] recently demonstrated that the leucine-rich repeat (LRR) domain senses salt stress with cell-wall receptors for plants to regulate growth under salt stress. Meanwhile, the Ca^2+^ concentration of cells increases because of the salt stress [26]. Thus, Ca^2+^ signals are responsive to the osmotic stress of the cells, and the signals are further transmitted to the nucleus by cGMP, MAPKKK, and MAPK to respond to salt stress (Data S4, signal). In the present study, the LRR protein (Data S4) was gradually upregulated with increasing salt concentration. Therefore, the LRR protein first senses salt stress and then transmits the signal with the Ca^2+^ signal.

#### 2.4.3. Photosynthetic C Precursors

**Figure 5:**
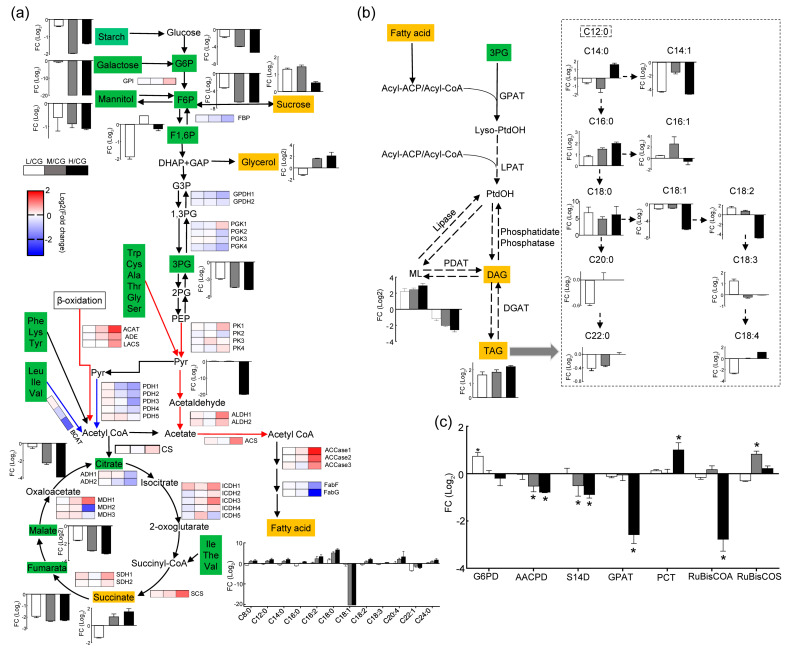
Metabolomic and proteomic features of central C metabolism in *Monoraphidium dybowskii* LB50 under NaCl concentrations. C metabolism (**a**), Kennedy pathway (**b**), and fold change in treatments relative to the control in G6PD, AACPD, S14D, GPAT, PCT, RuBisCOA, and RubisCOS (**c**). The green box indicates a decrease in metabolites; the yellow box indicates an increase in metabolites (compared with the control). Metabolic steps are represented by arrows. The red and blue arrows depict enzymes that are upregulated and downregulated under NaCl stress. G6P, glucose 6-phosphate; F6P, fructose-6-phosphate; F1,6P, fructose-1,6-bisphosphate; DHAP, dihydroxyacetone phosphate; GAP (G3P), glyceraldehyde 3-phosphate; 1,3PG, 1,3-bisphosphoglycerate; 3PG, 3-phosphoglycerate; 2PG, 2-phosphoglycerate; PEP, phosphoenolpyruvate; Pyr, pyruvate; ML, membrane lipid; GPAT, glycerol-3-phosphate acyltransferase; G6PD, glucose 6-phosphatedehydrogenase; AACPD, Acyl-ACP desaturase; S14D, Sterol 14 desaturase; PCT, RuBisCOA, RuBisCO activase; and RubisCOS, RuBisCO small subunit. The full names of the corresponding enzymes are provided in Data S4. L/CG, 20 g L^−1^ NaCl concentration compared to 0 g L^−1^ NaCl concentration; M/CG, 40 g L^−1^ NaCl concentration compared to 0 g L^−1^ NaCl concentration, and H/CG, 60 g L^−1^ NaCl concentration compared to 0 g L^−1^ NaCl concentration. Asterisks indicate statistically significant differences compared with the control conditions (*p* < 0.05).

The biophysical analysis of the kinetics of variable chlorophyll fluorescence revealed that cells showed no influence on the photosynthetic energy conversion efficiency of cells at L conditions but showed low efficiency in PSII under M and H conditions, as determined from Fv/Fm, proteins in PSI and PSII, and the cytochrome b6/f complex (Figure 4). Ribulose bisphosphate carboxylase oxygenase (Rubisco) activation was also remarkably downregulated only under H conditions. A small Rubisco subunit was upregulated under M conditions, whereas the light reaction was downregulated (Figure 5). Therefore, photosynthesis is not affected under L but is significantly decreased under M and H conditions. Cells synthesize additional pyruvate for lipid biosynthesis by boosting photosynthetic C fixation and a spot of carbohydrate degradation under L conditions. The electron carriers (ferredoxin-NADP+ reductase and ferredoxin (Data S1)) were downregulated gradually with increasing salt concentration, indicating that the electron transport was impaired. Meanwhile, according to the result of the ETR, cyclic electron flow played a key role in ATP supply under M and H conditions.

Proteomic and metabolomic analyses showed that enzymes and metabolites of the glycolytic pathway did not change significantly under L conditions (Figure 4). Glucose-6-phosphate dehydrogenase (G6PD), which is a key enzyme in the oxidative phosphate pentose pathway (OPPP), was upregulated only under L conditions (Figure 5c). The carbohydrates that were also identified in the OPPP were all downregulated (Data S4 and Figure 4a). Carbohydrates were degraded through the OPPP under L conditions. Under M and H conditions, pyruvate kinase (PK) was upregulated in the glycolytic pathway. Glyceraldehyde-3-phosphate dehydrogenase (GPDH), fructose bisphosphate aldolase, and fructose bisphosphatase (FBP) were downregulated, whereas phosphoglycerate kinase subunit (one protein only) was upregulated (Figure 5). Metabolomic analysis results showed that numerous carbohydrates decreased after NaCl stress. In particular, one of the key FBPs was downregulated with increasing salt concentration in gluconeogenesis, indicating that glycolysis was upregulated after salt stress. The gluconeogenesis pathway was reduced, and the synthesis of soluble sugar and glycerol were promoted (Figure 5a). Therefore, carbohydrates are degraded through the glycolysis pathway under M and H conditions.

#### 2.4.4. Pyruvate and Acetyl-CoA Synthesis

Pyruvate was derived from other sources, such as amino acid degradation. Most of the amino acids increased gradually with increasing NaCl concentration. Under L conditions, proline, histidine, tryptophan, and glutamine increased, whereas under M conditions, alanine and leucine increased. Under H conditions, all identified amino acids decreased (Figure 5). Therefore, amino acids are degraded for pyruvate synthesis. Many enzymes that synthesize amino acids were downregulated to cut block amino acid synthesis. 

Acetyl-CoA, which links glycolysis and FA synthesis, was mainly derived from pyruvate catalysis via pyruvate dehydrogenase (PDH) [27] and the PDH bypass pathway, where acetic acid was synthesized by aldehyde dehydrogenase (ALDH). Then, acetyl-CoA was synthesized by acetyl-CoA synthetase (ACS, Figure 5a). Acetyl-CoA was also synthesized by branched-chain amino acid (BCAA) degradation [28]. The branched-chain amino acid transaminase and methylcrotonoyl-CoA carboxylase, which degraded BCAA, decreased gradually with increasing salt concentration. ACS and ALDH were upregulated only under H exposure, whereas PDH was not upregulated. Acetyl-CoA was synthesized only by PDH bypass under H conditions.

#### 2.4.5. FA Synthesis and β-Oxidation

In FA synthesis, three ACCase proteins were identified; these proteins were upregulated only under H conditions and increased by 3.2-, 2.5-, and 2.5-fold. ACCase did not significantly change (Figure 5) under L and M conditions. FabF and FabG were significantly downregulated only under H conditions, further illustrating that lipid and TAG accumulation did not depend on the upregulation of the FA synthesis and Kennedy pathway but on the degradation of other macromolecules, resulting in C distribution and reduction (Figure 5b). However, lipid accumulation was induced by enhanced FA synthesis under H conditions.

FA degradation (β-oxidation) is a process involving energy extraction from lipids. In this process, acetyl-CoA can be produced and recycled via the TCA cycle or converted into carboxylic acid, which is decomposed into CO_2_ that is involved in biosynthesis via the noncyclic flux mode [29]. Succinic, glyoxylic, and malic acids can be synthesized in glyoxysome after FA degradation into acetyl-CoA. Acetyl-CoA C-acyltransferase, acyl-CoA dehydrogenase, and long-chain acyl-CoA synthetase, which were associated with β-oxidation, were upregulated under M and H conditions during lipid accumulation (Figure 5). Isocitrate lyase (Data S2), a key enzyme in the glyoxylate cycle, was also upregulated with increasing salt concentration. This expression pattern suggested that β-oxidation was activated under M and H conditions to produce additional energy for cell survival.

#### 2.4.6. TCA Cycle and Energy Sources

The identified metabolites involved in the TCA cycle decreased, except for succinate. Nearly 80% of the enzymes that were involved in the TCA cycle were upregulated (Figure 5 and Data S4). Thus, the TCA cycle can be enhanced through salt stress to obtain additional NADH and GTP/ATP. Under M and H conditions, succinyl-CoA synthase was significantly upregulated, and substrate phosphorylation was accelerated to produce additional GTP/ATP. Under L conditions, isocitrate dehydrogenase did not significantly change, indicating that cells did not rely on the accelerated TCA cycle to obtain NADH and GTP/ATP under L conditions. The changes in enzymes and proteins involved in electron transport and oxidative phosphorylation were not significant under L conditions (Figure 4b). Proteins were remarkably upregulated, including several subunits of complex I, complex II, complex III, and complex IV under M conditions. NADH-dehydrogenase (NDH, complex I) and succinate dehydrogenase (complex II) were upregulated under H exposure. Therefore, the accelerated TCA cycle and enhanced oxidative phosphorylation ensure the adequate supply of ATP to sustain survival and reproduction under M and H conditions.

Figure 5a suggests that TAG synthesis occurs by recycling C through the degradation of membrane glycerolipids via FA β-oxidation. Amino acid degradation can also increase C flow into the intermediate metabolism of the TCA cycle. Therefore, the upregulation of the TCA cycle, FA β-oxidation, and ML remodeling suggested that the C backbone can be reallocated to increase the FA and TAG accumulation.

### 2.5. Cell Cycle and Life Cycle to NaCl Concentrations

**Figure 6:**
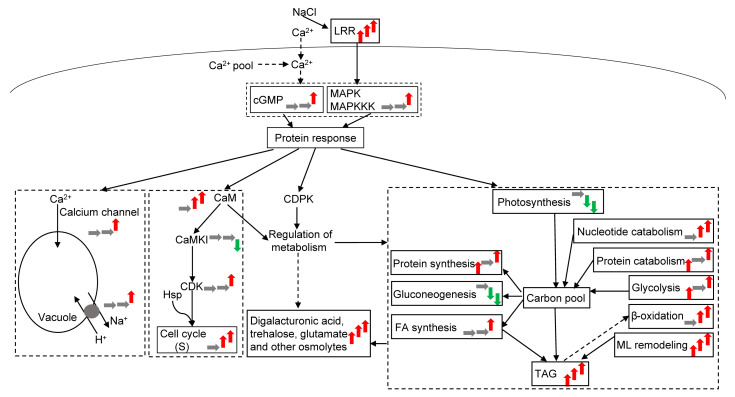
Working model for *Monoraphidium dybowskii* LB50 under different NaCl concentrations. Red and green arrows depict pathways, metabolites, or enzymes that are up- and downregulated under NaCl stress compared with the control. Gray arrows depict no changes compared with the control. Arrows denote L (20 g L^−1^ NaCl concentration), M (40 g L^−1^ NaCl concentration), and H (60 g L^−1^ NaCl concentration) from left to right compared to CG (0 g L^−1^ NaCl concentration).

Calprotectin and calmodulin (CaM) did not significantly change under L conditions but were significantly upregulated. When subjected to H exposure, the Ca^2+^ concentration was transported to the vacuole by the Ca channel (Figure 6). Meanwhile, proton transport and H^+^-ATPase were upregulated to cope with intracellular ion toxicity. In response to osmotic stress, the CaM-like domain protein kinases (CaMKIs) are downregulated under high salt stress by CaM. CaMKIs acted directly on the Ca^2+^/CaM pathway and participated in cyclin regulation. Heat shock proteins were upregulated under H conditions, which regulated the cell cycle and stopped most cell cycles in the S phase under H conditions. According to the flow cytometry results, the cell cycle did not significantly change under L and M conditions, but the number of cells in the S phase significantly increased under H conditions (Table S2, Figure 6). Freshwater algae can regulate the cell cycle to respond to different salt concentrations, and the life cycle of microalgae changes under H conditions. At the same time, Ca-dependent protein kinases and CaM regulate the metabolism of substances by the phosphorylation of phosphorylase.

## 3. Discussion

### 3.1. Osmotic Regulator

The cellular responses to salt stress are different from other conditions (especially nutrition deficiency, such as N deficiency) because of the ion effect and osmotic stress [30,31]. In resisting osmotic stress, many substances (mainly penetrating agents) are accumulated. Particularly, the increase in phospholipids and the change in FAs are significant [31,32]. The cells are obtained via global metabolomics and live to tolerate osmotic stress. According to the results obtained from physiology and biochemistry and integrated omics studies, a salinity-driven metabolic pathway of freshwater organisms was proposed to adapt to varying salt stress degrees (Figure 6).

In addition to common osmolytes, such as trehalose and glutamate, an increase in the accumulation of other osmolytes was an important phenomenon in restoring osmotic equilibrium. Digalacturonic acid was the most upregulated after salt stress;it increased from 14-fold to 18-fold. Digalacturonic acid was the main component of pectin, and the main component of the CW of green algae was pectin [33]. Digalacturonic acid has high antioxidant activity; it can regulate lipid metabolism and prevent membrane permeability, protecting the cell membrane and significantly improving the survival rate of damaged cells [34,35]. With its structure, digalacturonic acid may be an osmotic substance. The amino acids of the N-acetyl group also increased significantly, but the increasing amplitude of the glycerol was small. The osmotic regulators were similar to marine strains [5] but different from *Dunaliella* (Table S3). Freshwater microalgae retained the osmotic regulation function of marine organisms in response to salt stress.

### 3.2. Cell Membrane and Wall Response to NaCl Stress

The cell wall is the first barrier of life subjected to salt stress. Leucine-rich repeat extensins are a group of cell-wall proteins that harbor an N-terminal LRR domain and a C-terminal extension domain. The LRR domain is capable of sensing salt stress [25], implicating the involvement of the cell wall in salt stress perception and response. Algal cell walls are composed of various polysaccharides. In this study, LRR protein (Data S4) was gradually upregulated with increasing salt concentration, confirming this finding. Additionally, most of the proteins related to the cell wall were downregulated under H conditions. Among the upregulated proteins, UDP-glucose-4-epimerase (Data S4) plays a role in the interconversion between UDP-galactose and UDP-glucose. UDP-galactose serves as the precursor for the synthesis of MGDG, a component of plant cell walls and cell membranes. Therefore, these findings suggest that salt stress affects the cell wall and leads to various reactions in membrane lipids and carton pools.

Phospholipids play an important role in osmotic stress response [36]. The main adaptive changes in membrane lipids in response to an increase in salt concentration increased in PG and/or cardiolipin and matched with a corresponding fall in the PE (Table S3). PC and PG increased under NaCl stress. PE and PI mainly increased under L and M conditions, whereas PC mainly increased under H exposure. Therefore, *M. dybowskii* LB50 relied on two strategies to remodel the MLs and protect cells under NaCl stress: (1) increasing the structural lipids (phospholipids) and reducing the photosynthetic lipids (MGDG and DGDG) and (2) increasing the FA saturation and extending the long-chain FAs. These results were similar to those of the halophilic microalgae *Dunaliella* (Table S3).

Studies have shown that TAG accumulation in microalgae may rely on ML remodeling [37,38,39]. The remodeling process mainly depended on PDAT or acetyl-CoA and was derived from ML degradation and then re-entered into lipid synthesis [40,41]. TAG increased gradually with increasing NaCl concentration. This result may be due to the degradation of other lipid classes (glycolipids) via β-oxidation (Figure 5a). The glycolipids (i.e., DGDG and MGDG) of *M. dybowskii* LB50 were mainly transformed under NaCl stress. Bromke, Giavalisco, Willmitzer, and Hesse [31] found that when *Thalassiosira pseudonana* is cultured under N-limited conditions, DAG and TAG increase, whereas SQDG, PE, PC, and PG in polar lipids significantly decrease. However, under high NaCl concentrations, DAG, MGDG, PE, and PC decreased, but not PG, indicating that ML remodeling is inevitable under stress conditions, resulting in their conversion into TAG. The remodeling mechanism of the ML varied under different stress conditions. Photosynthetic glycolipids declined and phospholipid performance varied under different stresses, especially under osmotic stress.

### 3.3. Lipid and TAG Accumulation to NaCl Concentrations

Many metabolic activities eventually point to TAG, resulting in a large accumulation of TAG under salt stress (Figure 6). At the same time, TAG and lipids accumulated under salt stress [42,43], indicating that TAG accumulation played certain biological roles under salt stress. TAG accumulation mainly occurred in three ways. The first way involves CO_2_ fixation, leading to TAG accumulation (de novo synthesis). The second one involves the degradation of starch (carbohydrate) or amino acid, leading to TAG accumulation (intracellular material transformation). The third one involves a potential transformation of the MLs [44]. 

Although a large amount of TAG is accumulated under NaCl stress, the increment of TAG or lipids may be less than those under other conditions (especially N deficiency) due to the uniqueness of salt stress [45]. The mechanism of TAG or lipid accumulation under NaCl stress, in which phospholipid and osmolyte levels increased to protect cells, differed from other conditions, where many amino acids (proteins) and phospholipids were degraded [41,46,47]. The FA and TAG syntheses were upregulated due to the ion and the osmotic effects [48]. Considerable differences were also observed in TAG accumulation under different NaCl concentrations (Figure 6). Under L conditions, the glycolysis pathway increased. Photosynthetic C fixation was also not affected. Few remodeled glycolipids and phospholipids were converted into TAG, indicating that TAG or lipid accumulation was the comprehensive result of photosynthetic CO_2_ fixation and the degradation of a small amount of carbohydrate, leading to the allocation of C and reductant into lipids rather than lipid or TAG upregulation. Under M conditions, photosynthesis was hindered, glycolipids and phospholipids were remodeled and transformed TAGs (32%), and FA β-oxidation began to increase. The mechanism under M conditions depended on the energy from cyclic photophosphorylation, oxidative phosphorylation, FA β-oxidation, and C and reductant allocation from macromolecule degradation, but did not rely on photosynthetically fixed C (Figure 6). Under H conditions, the FA synthesis pathway was upregulated, and β-oxidation was upregulated markedly, indicating that an increment in FA β-oxidation would decrease the lipid productivity of the culture.

TAG accumulation is correlated with C metabolism [49,50]. The most important pathway in C metabolism is the glycolysis pathway, which provides the precursor (pyruvate) for TAG synthesis. In the work of Cheng, Feng, Zhang, Huang, Cheng, and Zhang [48], pyruvate is obtained via the glycolytic pathway, where only PK, GPDH, and phosphoglycerate mutase are upregulated in the culture of *Nitzschia* sp. under different salt concentrations. By contrast, Chang et al. [51] found that the expression levels of many enzymes involved in glycolysis in *Neodesmus* sp. UTEX 2219-4 does not significantly change under salt stress. Only branched chain starch enzymes are upregulated. In this case, few enzymes are upregulated in the glycolysis pathway. This finding was different from that observed under other stress conditions, in which many enzymes were upregulated during TAG accumulation. The cells were also subjected to L-catabolized carbohydrates depending on the OPPP. The cells under M and H conditions were dependent on the glycolysis pathway to catabolize carbohydrates. 

Acetyl-CoA, which is synthesized by PDH, PDH-bypass, and amino acid metabolism, is also a precursor for TAG synthesis [28,48,52]. Acetyl-CoA can also be synthesized from ACS and PDH in N-depletion conditions [53]. The availability of multiple sources for acetyl-CoA synthesis increases the amount of acetyl-CoA in response to different stress conditions, ensuring lipid and TAG accumulation. However, acetyl-CoA was synthesized only by PDH-bypass under H conditions because ACS and ALDH were upregulated only under H conditions in this pathway. By contrast, ALDH and ACS did not remarkably change under L and M conditions. Thus, acetyl-CoA does not accumulate under L and M conditions.

ACCase was upregulated only under H conditions, and other enzymes were downregulated in this pathway, suggesting that lipid accumulation in *M. dybowskii* LB50 did not depend on the upregulation of the FA synthesis pathway under L and M conditions. Lipid and TAG accumulation did not rely on the upregulation of the FA synthesis genes but on C distribution and reduction [49,54]. The enzymes in the FA synthesis pathway and genes involved in TAG synthesis did not significantly increase under L and M. Thus, the subsequent accumulation of TAGs and lipids under L and M conditions may be a consequence of C and reductant allocation and ML remodeling rather than a consequence of lipid and TAG biosynthesis upregulation, whereas the subsequent TAG and lipid accumulation under H conditions upregulated FA biosynthesis. 

FA β-oxidation is a process by which energy, acetyl-CoA, and CO_2_ are extracted from lipids. FA is degraded into acetyl-CoA. Then, succinate, malate, and glyoxylate are synthesized to participate in metabolic pathways through the glyoxylate cycle. FA β-oxidation is respired rather than serving as gluconeogenic substrates through the glyoxylate cycle [55]. The primary C source, that is, acetate, is no longer converted into cell building blocks through the glyoxylate cycle and gluconeogenesis but rather funneled directly into FA biosynthesis [56,57]. The FA degradation pathway is upregulated for cell reproduction and growth [58] or TAG accumulation [41]. Our proteomic data revealed that the enzymes involved in β-oxidation were highly upregulated. Meanwhile, the cell cycle was arrested at the S phase under H conditions. Therefore, salt stress activates the β-oxidation pathway to utilize energy from the lipid and TAG degradation for cell survival.

### 3.4. Energy for FA Synthesis

Large amounts of energy and reductants were required for FA synthesis and for the cells to resist osmotic stress. ATP production was supported by cyclic electron transport [51]. Lu et al. [59] found that NADPH from the OPPP drives the operation of cyclic electron flow around PSI in high-intertidal macroalgae under severe salt stress. In the present work, ATP production was produced based on photosynthetic phosphorylation under L conditions and cyclic electron transport because of the impaired photosynthetic linear phosphorylation with increasing NaCl concentration. The cyclic electron transport activity was enhanced when photosynthetic cells were subjected to environmental stress [60,61]. However, cyclic electron transport alone was insufficient to enhance lipid biosynthesis under M and H conditions, and other pathways were needed to acquire the required energy. Peng et al. [62] found that the complexes involved in oxidative phosphorylation (respiratory chain) are upregulated, and then, additional ATPs are obtained for growth and anabolism in *Coccomyxa subellipsoidea* C-169. In the present work, most of the complexes involved in photosynthesis were downregulated under M and H conditions, whereas respiratory complexes were upregulated. This finding indicated that energy was derived through oxidative phosphorylation, especially under M and H conditions.

Proteomic data showed that the number of DEPs involved in energy metabolism increased, and the number of upregulated DEPs involved in energy metabolism increased with salt concentration (Figure S2), indicating that a considerable amount of energy was consumed to resist osmotic stress. Photorespiration was weak, and respiration decreased first and then increased (oxidative phosphorylation). Carbohydrate degradation was insufficient to provide the energy needed to resist salt osmotic stress. FAs also began to degrade (under M and H conditions), providing additional energy. The energy production under L initially relied on photophosphorylation and the OPPP. With increasing NaCl concentration, energy was supplied by cyclic electron transport and oxidative phosphorylation. Energy was also produced by FA β-oxidation and substrate phosphorylation.

### 3.5. Cell Cycle and Life History

When subjected to stress, an organism alters its life cycle to cope with adversity. For example, the astaxanthin accumulation and nonmotile cell formation in *Haematococcus pluvialis* occur when culture conditions are unfavorable for growth and reproduction [63]. Plants can also reprogram the gene expression governed by transcriptional factors, histone acetylation, DNA methylation, and cytoskeleton expression in response to salt stress. For instance, certain transcription factors (such as MYB, GmMYB76, or GmMYB177 in Arabidopsis, or OsMYB2 in rice) can inhibit cell development and cell cycle and redirect energy toward stress resistance [64]. In maize roots, salt stress induces changes in histone acetylation in the promoter region of cell cycle genes, which can confer resistance to salt stress [65]. Furthermore, salt-induced alterations in DNA methylation (rice, *Oryza sativa*) can be maintained through mitotic cell division [66]. Additionally, the cytoskeleton plays a crucial role in salt stress response, particularly in cell division and growth. In this study, *M. dybowskii LB50* is a unicellular plant, which can also inhibit salt stress through cell division and the cell cycle. In many plant species, TAGs are the major storage lipids, serving as important energy reserves in seeds for subsequent germination and seedling development. TAGs are also essential for pollen development and sexual reproduction [67,68,69,70]. In the present study, the cells increased in the S phase of the cell cycle, where DNA was synthesized (Table S2). The cells did not grow and began to multiply under H conditions. At the same time, β-oxidation was intensified. These findings suggested that TAG accumulation served as the primary energy repository to generate the next generation and organism-regulated cell cycle or life history to respond to high salt stress.

## 4. Materials and Methods

### 4.1. Organisms and Experiment

*M. dybowskii* LB50 used in this study was provided by XuDong Xu of the Key Laboratory of Algal Biology, Institute of Hydrobiology (IHB), Chinese Academy of Sciences (CAS). The stock cultures were maintained in a sterilized BG11 medium. *M. dybowskii* LB50 was cultivated in a modified BG11 medium consisting of 0.25 g L^−1^ urea and 0.1 M NaHCO3 in 400 mL of a culture medium in a 500 mL flask [8]. Different amounts of NaCl were added to prepare 0 g L^−1^ (control group, CK), 20 g L^−1^ (low NaCl concentration, L), 40 g L^−1^ (medium NaCl concentration, M), and 60 g L^−1^ (high NaCl concentration, H) solutions. The three cultures of each treatment were grown with continuous illumination at 60 μmol m^−2^ s^−1^ at 25 ± 1 °C. The culture flask underwent continuous bubbling with filter-sterilized air (from the bottom) through a transparent glass tube. For physiological and biochemical characteristics analyses, the sample was harvested at five different points of growth phase (day 0, 1, 2, 3, and 4) from each culture flask. For FAs, metabolite profiling, and proteomic analyses, four sampling points on day 1 were chosen to investigate the expression. Three independent cultures and one mixed sample per condition were used for metabolomic studies plus two cultures per condition for proteomics on day 1.

### 4.2. Determination of Physiological Characteristics

A total of 4 mL of cell culture was harvested to determine the algal density and dry weight, as described by Yang et al. [8]. To determine the physiological and biochemical characteristics (carbohydrates, soluble sugars, starch, trehalose, chlorophyll, and protein), we harvested 1 mL of sample cells by centrifuging each indicator. Carbohydrates and soluble sugars were quantified using the phenol sulfuric acid method [71,72]. The absorbance was determined at 490 nm in a UV-2000 spectrophotometer (Beijing Purkinge General Instrument Co., Ltd., Beijing, China). Starch was assayed through an enzymatic method [73]. Hydrolysate-containing glucose released from starch was determined at 520 nm. Trehalose was quantified following the anthrone sulfuric acid colorimetric method [74]. Chlorophyll was extracted using 95% ethanol at 4 °C overnight according to the method described by Jeffrey and Humphrey [75]. Total protein was determined using the Bradford method, with bovine serum albumin as the standard [76].

The cell culture (2 mL) was harvested for its photochemical fluorescence efficiency, non-photochemical quenching (NPQ), and cell cycle. The photochemical fluorescence efficiency (Fv-to-Fm ratio, Fv/Fm) of PSII and NPQ were measured using a plant efficiency analyzer (PEA, Hansatech^®^, King’s Lynn Norfolk, UK). The original fluorescence (Fo) was determined under the irradiance of measuring light. A saturation pulse was applied to obtain maximum fluorescence (Fm) in the dark-adapted samples. A saturating pulse was applied to obtain a stationary level of maximum fluorescence (Fm’). ETRs were estimated using a Dual-PAM-100 Fluorometer (Walz, Effeltrich, Germany). The cell cycle was detected by flow cytometry (BD FACSAriaTM III, San Jose, CA, USA).

The effective PS II quantum yield was calculated as follows:

Fv/Fm = (Fm − Fo)/Fm; NPQ = (Fm − Fm’)/Fm’. 

The ETR was calculated as follows:

ETR = [(Fm’ − F)/Fm′] × PAR × 0.84 × 0.5. 

where F is the fluorescence yield observed in the illuminated samples before applying the saturation light pulse, which is normally higher than Fo. PAR is the amount of photosynthetically absorbed radiation in the chlorophyll fluorimeter, 0.84 is the cell-specific absorption coefficient, and 0.5 is derived from the assumption that the radiant energy is equally divided between PS I and PS II.

### 4.3. Lipid Extraction and FA Analysis

Cultures (40 mL) containing at least 50 mg of microalgal dry weight were harvested for lipid extraction. The total lipid was extracted as described by Yang et al. [8]. The lipids dissolved in the lower chloroform phase were spotted on thin-layer chromatography (TLC) plates [77]. Neutral lipids were separated through TLC using hexane/ethyl nether/acetic acid (70/30/1, *v*/*v*/*v*). The polar lipids were separated through TLC using acetone/toluene/water (91/30/7.5, *v*/*v*/*v*) [78,79]. The lipids separated by TLC can be stained with sulfuric acid, iodine, an α-naphthol stain for GL, and a molybdenum blue spray reagent for PL [80].

After visualization and identification, the lipid bands were immediately and carefully scraped out, and the FAs of the total lipid and lipid fractions were analyzed by GC after direct transmethylation with H_2_SO_4_ in methanol. The FA methanol esters (FAMEs) were extracted and analyzed as described by Yang et al. [8] (refer to Methods S1 for details).

### 4.4. Metabolite Profiling in M. dybowskii LB50

A total of 50 mL of the cells were quenched and extracted for metabolite analysis according to Lu et al. [81] with modifications. The cells were sprayed onto 60% (*v*/*v*) cold (−40 °C) methanol to arrest metabolism instantaneously and then centrifuged at 4000 rpm for 4 min. The cell samples were lyophilized by a freeze dryer. A total of 60 mg lyophilized samples were extracted by an extraction buffer containing the internal standard, followed by derivatization and injection for GC/MS analysis by an Agilent 7890B GC system coupled to an Agilent 5977A MSD system (Agilent, Santa Clara, CA; refer to Methods S1 for details).

The acquired MS data from GC/MS were analyzed by Chroma TOF software (version 4.34, LECO, St Joseph, MI, USA). Metabolites were qualitative and aligned with the statistical comparison component by the NIST, Fiehn, GOLM, and Massbank databases. Internal standards and any known pseudo-positive peaks were removed from the data set, and the peaks from the same metabolite were combined. The resulting data were normalized to the total peak area of each sample and imported into a SIMCA (version 14.0), where principal component analysis (Figure S3), partial least-squares discriminant analysis (PLS-DA), and orthogonal PLS-DA (OPLS-DA) were performed. The differential metabolites were selected based on the combination of a statistically significant threshold.

### 4.5. iTRAQ Proteomic Analysis

Culture (2 mL) was harvested, resuspended into PBS, and then centrifuged at 4000× *g* for 2 min. The cell pellets were resuspended in lysis buffer to extract the proteins. The proteins were reduced, alkylated, digested with trypsin, and labeled using 8-plex iTRAQ, according to the manufacturer’s recommendations (AB Sciex Inc., Framingham, MA, USA, http://sciex.com/, accessed on 9 October 2019). Eight samples (two biological replicates for four samples) were labeled with different iTRAQ tags.

Peptides from each group were mixed and resolved into 15 fractions through HPLC, followed by Q Exactive MS (qThermo Fisher Scientific, San Jose, CA, USA). The resulting MS/MS data were searched against a Uniprot Monoraphidium neglectum protein database using MaxQuant (version 2.3.02), with a false discovery rate of 1%. A functional annotation was attributed to the quantified proteins through gene ontology (GO) annotation using Blast2GO 5 PRO software (http://www.blast2go.de, accessed on 9 January 2020) [82], Kyoto Encyclopedia of Genes and Genomes pathway analysis, and GO functional enrichment analysis. Two technical replicates with tag swapping were conducted for each biological replicate. The proteins with 1.5-fold change and Q < 0.05 were considered DEPs, which must be defined in at least 1 replicate experiment (refer to Methods S1 for a complete description).

### 4.6. Statistical Analysis

Values are means ± SD of independent experiments. The paired-sample Student’s t-test and ANOVA were performed using the SPSS 19.0 package (SPSS, Chicago, IL, USA), and a *p*-value of 0.05 indicated statistical significance.

## 5. Conclusions

Microalgae can adopt a variety of strategies for generating a salinity tolerance mechanism, including increasing ion transport and osmolytes, adjusting cell cycle and life history, and accumulating TAG. The cells gradually adjust their cycle and life history to survive under high salt stress. Our data also suggested that TAG was used as a protective mechanism to help the cells survive under salt-stress conditions. Therefore, the cells need to coordinate whole life processes to adapt to increasing salt stress. The net effect of this intracellular reorganization was that cells could quickly respond to changes in external NaCl availability and further adapt to the environment.

## Figures and Tables

**Table 1:** Fatty acid composition of total lipids, TAG, DGDG, and PC in *Monoraphidium dybowskii* LB50 under NaCl stress.

		14:00	14:01	16:00	16:01	18:00	18:01	18:02	18:03	18:04	Others	SFA	MUFA	PUFA	DU
TL	CG	0.64	0.03	21.46	2.8	1.11	42.88	16.25	8.78	4.83	1.22	24.29	45.71	30.01	1.06
	L	1.08	0.06	41.87	4.26	1.65	12.18	23.6	12.4	ND	2.9	47.12	16.5	36.37	0.89
	M	1.35	ND	60.86	3.87	0.15	4.04	7.39	2.3	18.78	1.26	63.27	7.9	28.83	0.66
	H	2.33	0.86	78.78	ND	2.49	4.53	0.43	1.49	8.78	0.3	83.75	5.4	10.86	0.27
TAG	CG	0.69	1.57	19.36	0.09	0.23	64.69	1.64	2.05	3.23	9.69	20.28	66.35	3.68	0.74
	L	0.47	0.08	34.03	0.13	23.38	28.91	4.57	2.8	2.7	5.63	57.88	29.13	7.36	0.44
	M	0.29	0.56	54.04	0.54	6.6	32.4	2.87	0.3	ND	2.39	60.94	33.5	3.16	0.4
	H	2.09	0.06	76.49	0.06	15.88	1.04	0.06	0.03	3.88	4.3	94.46	1.15	0.09	0.01
DGDG	CG	3.69	ND	39.51	5.73	21.22	0.46	24.29	0.55	ND	4.55	66.18	6.19	27.63	0.61
	L	7.69	0.15	38.99	ND	11.1	32.14	7.62	0.27	0.67	1.36	58.34	32.29	9.37	0.51
	M	10.38	ND	38.71	ND	10.96	24.91	13.07	1.08	0.2	0.7	60.05	24.91	15.05	0.55
	H	8.2	5.1	3.64	14.33	12.11	5.35	7.56	12.57	4.25	26.89	27.99	24.77	47.24	1.19
PC	CG	7.84	ND	40.16	ND	14.64	18.84	5.13	2.62	3.83	6.95	63.6	18.84	17.56	0.54
	L	1.8	0.74	24.39	ND	20.8	35.44	4.51	0.96	ND	11.37	50	36.18	13.83	0.64
	M	1.35	ND	2.51	0.27	19.86	14.36	9.94	28.87	ND	22.86	36.26	14.62	49.12	1.13
	H	ND	0.25	1.42	1.81	69.72	2.29	2.14	1.11	10.53	10.72	78.99	4.35	16.66	0.38

TL, total lipid; TAG, triacylglycerol; DGDG, digalactosyldiacylglycerol; PC, phosphatidylcholine; SFA, saturated fatty acid; MUFA, monounsaturated fatty acid; PUFA, polyunsaturated fatty acid; DU, degree of unsaturation. CG, 0 g L^−1^ NaCl concentration; L, 20 g L^−1^ NaCl concentration; M, 40 g L^−1^ NaCl concentration, H, 60 g L^−1^ NaCl concentration. All the values are the mean of three independent determinations. ND, not detected.

## Data Availability

Data on metabolomics can be accessed via Metabolomics Workbench (https://www.metabolomicsworkbench.org, accessed on 29 August 2023) via datatrack ID #1662. Data on proteomics can be accessed via iProX (www.iprox.cn, accessed on 1 September 2023) via datatrack ID IPX0007014000. Other data can be found as stated previously.

## Supplementary Materials

The following supporting information can be downloaded at https://www.mdpi.com/article/10.3390/plants12193495/s1, Methods S1. Detailed methods; Figure S1: Electron micrographs of Monoraphidium dybowskii LB50 induced with NaCl for 1 day; Figure S2. GO and COG annotation of different expression proteins; Figure S3. Principal component analysis-derived score plots of the global metabolite profiles; Table S1. Quantity of annotated proteins; Table S2. Cell cycle of *Monoraphidium dybowskii* LB50 under different NaCl concentrations; Table S3. Compatible solutes and changes in the plasma membrane and fatty acids in microorganisms under salinity stress; Data S1. Identified metabolites in *Monoraphidium dybowskii* LB50 under different NaCl concentrations; Data S2. Identified proteins in *Monoraphidium dybowskii* LB50 under different NaCl concentrations; Data S3. Different expression proteins in *Monoraphidium dybowskii* LB50 under different NaCl concentrations; Data S4. DEPs and enzymes in pathways. References [83,84,85,86,87,88,89,90,91,92,93,94,95,96,97,98] are cited in the supplementary materials.
