# Supplementary material for: Strategies of NaCl Tolerance in Saline–Alkali-Tolerant Green Microalga Monoraphidium dybowskii LB50
Source: Plants (Basel). 2023 Oct 7;12(19):3495. doi: 10.3390/plants12193495 (PMC10575140; doi:10.3390/plants12193495)
Supplement: Supplementary file 1 [file plants-12-03495-s001.zip › Figures S-revised.docx]

**Short supporting information**

**Figure S1.** Electron micrographs and confocal microscopy of Monoraphidium dybowskii LB50 induced with NaCl for 1 d.

**Figure S2.** GO and COG annotation of different expression proteins.

**Figure S3.** Principal component analysis-derived score plots of the global metabolite profiles.


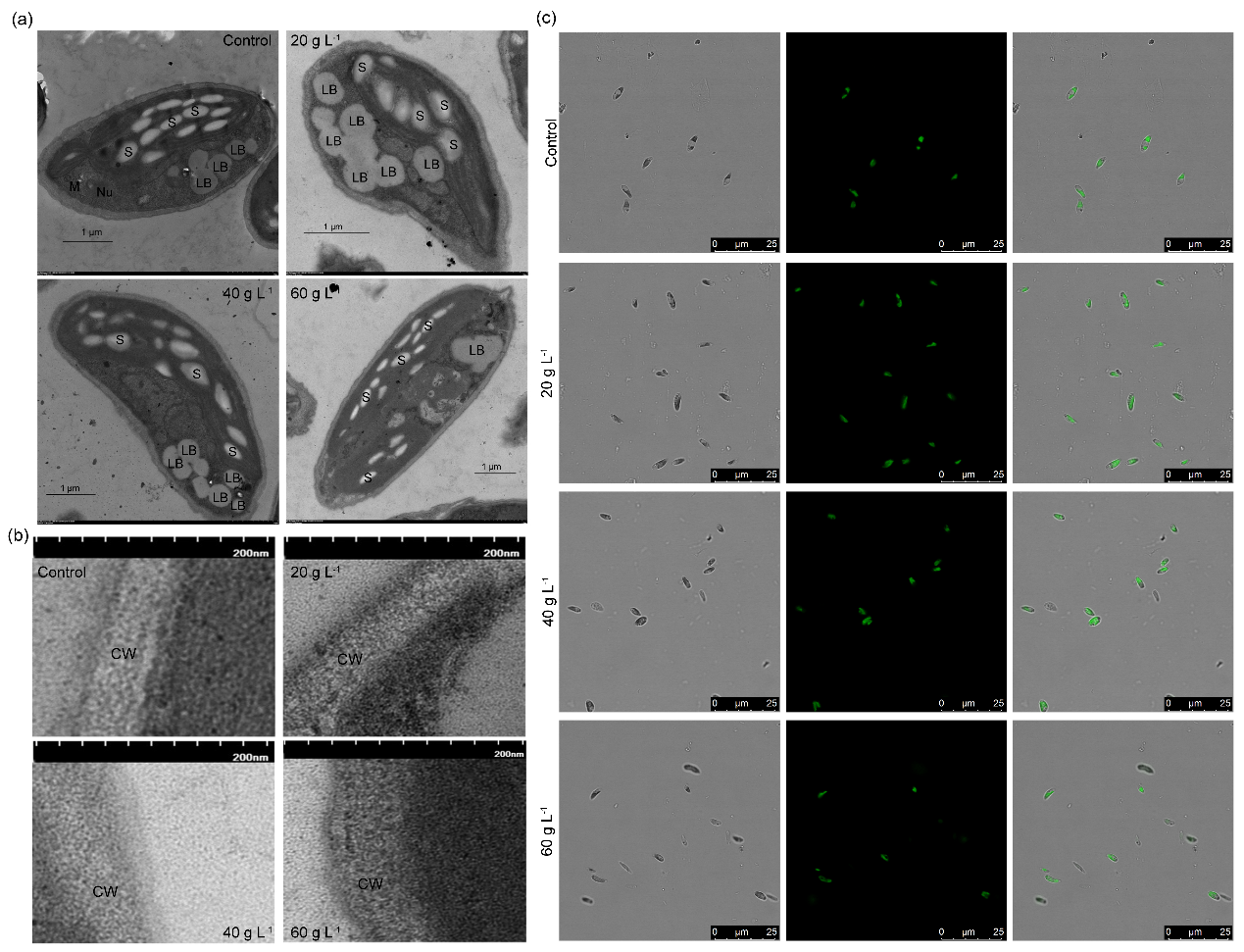


**Figure S1**. Electron micrographs and confocal microscopy of *Monoraphidium dybowskii* LB50 induced with NaCl for 1 d. Cell (a), Cell wall (b), and chloroplast morphology (c). LB, lipid body; Nu, nucleus; S, starch grains; Py, pyrenoid; V, vacuoles; CW, cell wall; M, mitochondria.


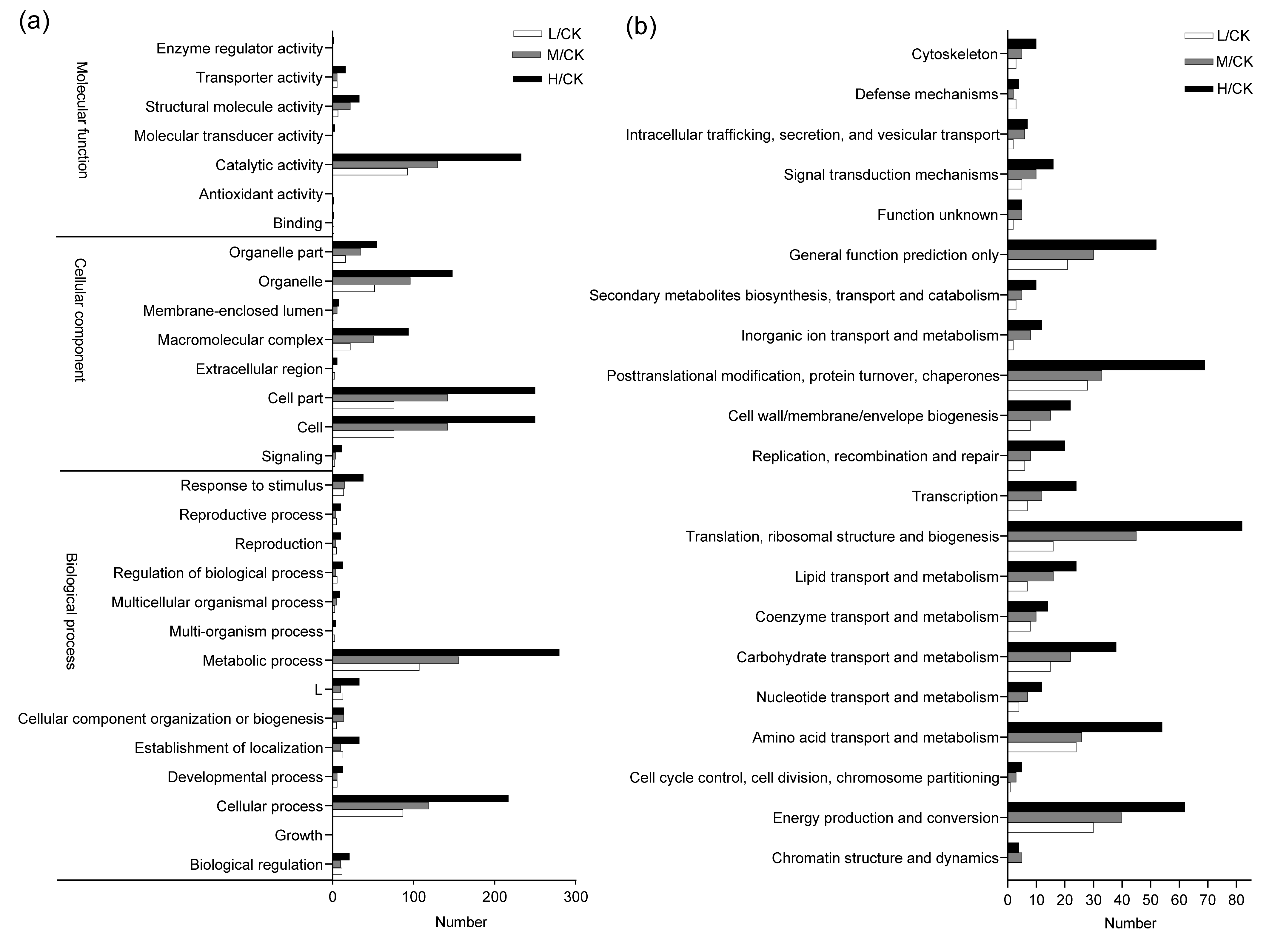


**Figure S2**. GO (a) and COG (b) annotation of different expression proteins. CK, 0 g L^−1^ NaCl concentration; L, 20 g L^−1^ NaCl concentration; M, 40 g L^−1^ NaCl concentration, H, 60 g L^−1^ NaCl concentration.


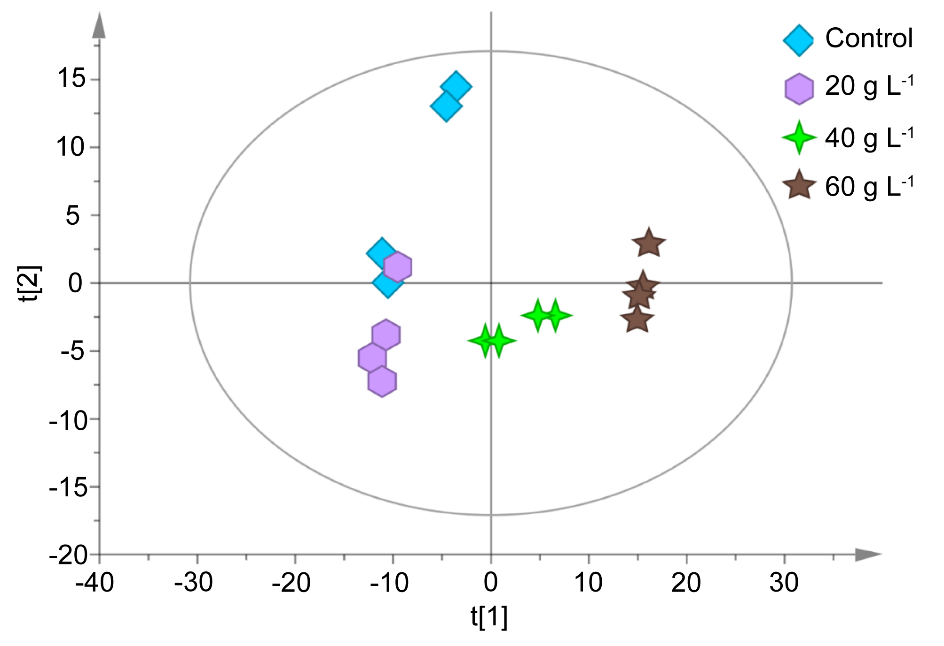


**Figure S3.** Principal component analysis-derived score plots of the global metabolite profiles.
