# Supplementary material for: Strategies of NaCl Tolerance in Saline–Alkali-Tolerant Green Microalga Monoraphidium dybowskii LB50
Source: Plants (Basel). 2023 Oct 7;12(19):3495. doi: 10.3390/plants12193495 (PMC10575140; doi:10.3390/plants12193495)
Supplement: Supplementary file 1 [file plants-12-03495-s001.zip › Methods S1-revised.docx]

**Methods S1.** Detailed methods

**Determination of the Physiological Characteristics**

The soluble sugars were extracted in 80% (v/v) alcohol heated to 80 °C in a water bath. The extract was then centrifuged at 5,000 g for 10 min. The supernatant was used for the estimation of soluble sugars concentrations. The reaction mixture consisted of 1 ml 5% phenol and 5 ml 98% H_2_SO_4_. Once the extract had cooled, its absorbance was determined at 490 nm, using glucose (99%) as the standard.

A total 2 ml of sample cells were harvested and resuspended in 80% (v/v) ethanol and incubated in a water bath at 80–85 °C for 5 min, in order to extract interfering compounds. Starch was hydrolyzed to liberate glucose by adding 30 units of amyloglucosidase enzyme in citrate buffer (pH 4.5) and incubated at 55 °C for 1 h.

Chlorophyll extraction used 95% ethanol at 4 °C overnight according to the method by Jeffrey and Humphrey [74]. Light absorbance of 665 nm and 649 nm were measured using a UV-2000 spectrophotometer (Beijing Purkinge General Instrument Co., Ltd.). Chlorophyll content was determined using the formula Chl *a* = 13.95A665 − 6.88A649, Chl *b* = 24.96A649 − 7.32A665, where for the total chlorophyll content (mg g^−1^DW) = Chl *a* + Chl *b*

**Transmission electron microscopy (TEM) and confocal microscopy**

The cells were fixed at 4 °C with glutaraldehyde (2.5 % in 0.2 M phosphate buffer, pH 7.6) for 12 h, post-fixed in 4 % OsO_4_ at 25 °C for 3 h. The materials were centrifuged, dehydrated in an acetone/water series, and embedded in resin. Ultrathin sections were stained with uranyl acetate and lead citrate. Micrographs were taken using transmission electron microscopy (H-7650, Hitachi, Japan). Chloroplast morphology of cellsk were taken using confocal microscopy (SP8, Leica, Germany).

**Lipid Extraction and fatty acid analysis**

The total lipid was extracted from approximately 50 mg the dried algae (w1) using a Soxhlet apparatus, with chloroform–methanol (1:2, v/v) as the solvent. The total lipid was transferred into a pre-weighted beaker (w2), and blow-dried in a fume cupboard. The lipid was dried to a constant weight in an oven at 105 °C and weighted (w3).

The lipid content (LC %) was determined according to the Eqs:

LC (%) = (w3- w2)/ w1×100

After visualization and identification, lipid bands were immediately and carefully scraped out, and fatty acids, total lipid, NL, GL and PL were analyzed by gas chromatography (GC) after direct transmethylation with sulphuric acid in methanol. The fatty acid methanol esters (FAMEs) were extracted with hexane and analyzed by GC mass spectrometry (GC/MS) (Thermo Scientific ITQ 700™, USA) equipped with a flame ionization detector (FID) and a fused silica capillary column (60 m×0.25 mm×0.25 μm; Aglient Technologies, USA). The injector and detector temperatures were maintained at 270 and 280 °C, respectively, with an oven temperature gradient of 50–170 °C at 40 °C min^−1^ after a 1 min hold time at 50 °C, then with an oven temperature gradient of 170–210 °C at 18 °C min^−1^ after a 1 min hold for 1 min. All parameters of the FAMEs were derived from the calibration curves generated from the FAME standard mix (Supelco 37 component FAME mix, Sigma–Aldrich, USA).

**Metabolites** **extraction in *M. dybowskii* LB50**

1 ml of extraction buffer [methanol: water (4:1, v/v), 200 μl of chloroform, 20 μl of 2-chloro-l-phenylalanine (0.3 mg ml^−1^) dissolved in methanol as internal standard] were added to the 60 mg of lyophilized cells for metabolites extraction. And then was subject to ultrasonication (500 w, 6 min) and extracted for 20 min in icewater bath. 500 μl of extraction supernatant was dried in a freeze concentration centrifugal dryer.

Derivatization of metabolites was accomplished via methoximation and trimethylsilylation. An 80 μl of methoxylamine hydrochloride (15 mg ml^−1^ in pyridine) was subsequently added to the dried sample and incubated at 37 °C for 90 min. After that, 80 μl of N, O-Bis (trimethylsilyl) trifluoroacetamide (with 1% trimethylchlorosilane) and 20 μl n-hexane was added into the mixture, which was vortexed vigorously for 2 min and then derivatized at 70 °C for 60 min. The supernatant was transferred to gas chromatograph vials prior to GC/MS analysis.

A DB-5MS fused- silica capillary column (30 m × 0.25 mm × 0.25 μm, Agilent J & W Scientific, Folsom, CA, USA) was utilized to separate the derivatives. The injector temperature was maintained at 260 °C. Injection volume was 1 μL by splitless mode. The initial oven temperature was 90 °C, ramped to 180 °C at a rate of 10 °C min^-1^ to 240 °C at a rate of 5 °C min^−1^, to 290 °C at a rate of 25 °C min^−1^, and finally held at 290 °C for 11 min. The temperature of MS quadrupole, and ion source (electron impact) was set to 150, and 230 °C, respectively. The collision energy was 70 eV. Mass data was acquired in a full-scan mode (m/z 50-450), and the solvent delay time was set to 5 min.

**Total protein extraction and** **iTRAQ proteomic analysis**

**Protein extraction and digestion**

At each point, cell culture (2 ml) was harvested and resuspended into PBS. The tube was centrifuged at 4,000 × g for 2 min. The cell pellets were suspended in 300 μL lysis buffer (7 M urea, 2 M thiourea, 4% CHAPS, 40 mM Tris-HCl, pH 8.5), and PMSF (1 mM, final concentration), EDTA (2 mM, final concentration) were added to the lysis solution with vortexing for 5 min, followed by dithiothreitol (DTT, 10 mM, final concentration) was add and mixed. The sample was centrifuged at 25,000×g for 20 min, and the supernatant was mixed with ice-cold acetone (1:9, v/v) containing 10 Mm DTT. After repeating the lysis step twice, the supernatants were combined, and proteins were harvested by centrifugation at 10,000×g for 30 min after precipitation at −20 °C overnight. The protein pellet was dissolved in 200 μl 0.5 M triethylammonium bicarbonate (TEAB) and total protein concentration was measured by Bradford Protein Assay Kit.

200 μg of protein from each lysate was reduced with 10 mM DTT at 56 °C for 1 hour, and then alkylated with 55 mM iodoacetamide for 45 min at room temperature in the dark. Ice-cold acetone (1:4, v/v) was added for 2 hours at -20 °C, the supernatant was discarded after centrifugation 25,000 x g for 20 minutes. The pellet was dissolved in 200 μl 0.5 M TEAB and sonicated for 15 minutes. Proteins was harvested by centrifugation 25,000 x g for 20 minutes. Proteins remaining on the filter were digested with trypsin at 37 °C for 4 h. After digestion, the peptide mixture was frozen at −20 °C to halt protease activity. Samples not immediately analyzed were stored at −80 °C.

**iTRAQ labling and LC–MS/MS proteomic analysis**

100 μg peptides were dried through vacuum centrifugation, reconstituted in 0.5 M TEAB, and processed according to the manufacturer’s recommendations for 8-plex iTRAQ. Eight samples (two biological replicates for four samples) were labeled with different iTRAQ tags.

The peptides labeled with respective is obaric tags, incubated for 2 h. Then the iTRAQ labeled peptides were fractionated using the Strong Cationic Exchange (SCX) chromatography. For SCX chromatography using the Shimadzu LC-20AB HPLC Pump system, the peptide from digestion is reconstituted with 4 ml buffer A (25mM NaH2PO4 in 25% ACN, pH 2.7) and loaded onto a 4.6 x 250 mm Ultremex SCX column containing 5-μm particles (Phenomenex). The peptides are eluted at a flow rate of 1 ml min^−1^ with a gradient of buffer A for 10 min, 5-35% buffer B (25 mM NaH2PO4, 1M KCl in 25% ACN, pH2.7) for 11 min,35-80% buffer B for 1 min. The system is then maintained in 80% buffer B for 3 min before equilibrating with buffer A for 10 min prior to the next injection. Elution is monitored by measuring absorbance at 214 nm, and fractions are collected every 1min. The eluted peptides are pooled as 20 fractions, desalted by Strata XC 18 column (Phenomenex) and vacuum-dried.

Each fraction was resuspended in a certain volume of solution A (2% ACN and 0.1% FA) and was loaded on a Shimadzu LC-20AD nano HPLC by the autosampler onto a 2 cm C18 trap column. Then, the peptides were eluted onto a 10 cm analytical C18 column (inner diameter of 75 μm) packed in-house. The samples were loaded at a rate of 8 μl min^−1^ for 4 min. Then, the 41-min gradient was run at a rate of 300 nl min^−1^ starting from 5% to 35% solution B (98% ACN and 0.1% FA), followed by a 5-min linear gradient to 80% solution B, maintenance at 80% solution B for 4 min, and return to 5% in 1 min.

The peptides were subjected to nanoelectrospray ionization followed by tandem mass spectrometry (MS/MS) in a Q Exactive (Thermo Fisher Scientific, San Jose, CA, USA) coupled online to the HPLC. Intact peptides were detected in the orbitrap at a resolution of 70,000. The peptides were selected for MS/MS using the high-energy collision dissociation operating mode with a normalized collision energy setting of 27.0; ion fragments were detected in the orbitrap at a resolution of 17,500. A datadependent procedure that alternated between one MS scan followed by 15 MS/MS scans was applied for the 15 most abundant precursor ions above a threshold ion count of 20,000 in the MS survey scan with a subsequent dynamic exclusion duration of 15 s. The electrospray voltage applied was 1.6 kV. Automatic gain control (AGC) was used to optimize the spectra generated by the orbitrap. The AGC target for full MS was 3×10^6^ and 1×10^5^ for MS2. For MS scans, them/z scan range was 350 to 2000 Da. For MS2 scans, them/z scan range was 100-1800.

**Protein data analysis**

The raw MS/MS data was converted into MGF format by thermo scientific tool Proteome Discoverer, and the exported MGF files were searched using Mascot version 2.3.02 in this project against the selected database (Uniprot *Monoraphidium neglectum* protein database). A functional annotation was attributed to the quantified proteins using gene ontology (GO) annotation using Blast2GO software (http://www.blast2go.de) (Conesa et al., 2005), Kyoto Encyclopedia of Genes and Genomes (KEGG) pathway analysis, functional (GO) enrichment analysis. Two technical replicates with tag swapping were also conducted for each biological replicate. Proteins with 1.5-fold change and Q-value less than 0.05 were determined as differentially expressed proteins. Final differentially expressed proteins must be defined in at least one replicate experiment.
