# Supplementary material for: Strategies of NaCl Tolerance in Saline–Alkali-Tolerant Green Microalga Monoraphidium dybowskii LB50
Source: Plants (Basel). 2023 Oct 7;12(19):3495. doi: 10.3390/plants12193495 (PMC10575140; doi:10.3390/plants12193495)
Supplement: Supplementary file 1 [file plants-12-03495-s001.zip › Tables S-revised.docx]

**Short supporting information**

**Table S1.** Quantity of annotated proteins.

**Table S2.** Cell cycle of *Monoraphidium dybowskii* LB50 under different NaCl concentrations.

**Table S3.** Compatible solutes, changes of the plasma membrane and fatty acids in microorganisms under salinity-induced.

Table S1. Quantity of annotated proteins

|  | 1.5-fold change | L | M | N |
| --- | --- | --- | --- | --- |
| All | Up-regulated | 98 | 141 | 215 |
|  | Down-regulated | 82 | 139 | 285 |
|  | All-regulated | 180 | 280 | 500 |
| Annotation | Up-regulated | 77 | 103 | 173 |
|  | Down-regulated | 66 | 119 | 214 |
|  | All-regulated | 143(80%) | 222(80%) | 387(77%) |

L, 20 g L^−1^ NaCl concentration campared to 0 g L^−1^ NaCl concentration; M, 40 g L^−1^ NaCl concentration campared to 0 g L^−1^ NaCl concentration, and H, 60 g L^−1^ NaCl concentration campared to 0 g L^−1^ NaCl concentration.

Table S2. Cell cycle of *Monoraphidium dybowskii* LB50 under different NaCl concentrations.

|  | CK | L | M | H |
| --- | --- | --- | --- | --- |
| Diploid (%) | 100.00 | 100.00 | 100.00 | 100.00 |
| Dip G1 | 69.97% at 41.30 | 98.54% at 49.68 | 98.69% at 39.71 | 18.64% at 33.06 |
| Dip G2 | 0.00% at 82.61 | 0.00% at 99.36 | 0.00% at 79.43 | 0.00% at 66.13 |
| Dip S (%) | 30.03 | 1.46 | 1.31 | 81.36 |
| G2/G1 | 2.00 | 2.00 | 2.00 | 2.00 |
| %CV | 22.15 | 23.88 | 29.10 | 15.82 |
| Total S-Phase (%) | 30.03 | 1.46 | 1.31 | 81.36 |
| Debris (%) | 1.36 | 3.58 | 0.00 | 9.26 |
| Aggregates (%) | 18.09 | 23.45 | 23.52 | 18.27 |
| Modeled events | 11077.00 | 10799.00 | 10696.00 | 10245.00 |
| All cycle events | 8922.00 | 7880.00 | 8188.00 | 7425.00 |
| Cycle events per channel | 211.00 | 155.00 | 201.00 | 218.00 |
| RCS | 6.09 | 6.88 | 4.71 | 1.45 |

CK, 0 g L^−1^ NaCl concentration; L, 20 g L^−1^ NaCl concentration; M, 40 g L^−1^ NaCl concentration, H, 60 g L^−1^ NaCl concentration.

Table S3. Compatible solutes, changes of the plasma membrane and fatty acids in microorganisms under salinity-induced.

| Microorganism | Osmolytes | Lipid/Fatty acids | Fluidity/Permeability | References |
| --- | --- | --- | --- | --- |
| Archaea: *Alkaliphilic halobacteria; Natronococcus* (Halobacteria) | Trehalose and 2-sulfotrehalose; glycine betaine uptake | Branched C20 and C25 lipids, maintenance of a highly negative charge surface density by a high concentration of acidic lipids |  | [83] |
| Bacteria: *Halomonas elongata; H. halophila* (Halomonadaceae) | Ectoine and hydroxyectoine or glycine betaine (uptake) | Increased anionic lipids (CL and moderate increase in PG), increase in neutral PC relative to PE. Decrease in branched-chain fatty acids. Increase in CFA, unsaturated fatty acids and GL | Increased fluidity | [84,5] |
| Bacteria: *Halorhodospira halochloris* (Ectothiorhodospiraceae) | Glycine betaine (synthesis and uptake), Ectoine, trehalose | Strong increase in PC, moderate increase in PG | Increased fluidity | [85] |
| Bacteria: *Halobacillus halophilus* (Bacillaceae, marine strains) | Proline, glutamine and glutamate, Ectoine, N-acetyl ornithine and N acetyl lysine |  |  | [19] |
| Bacteria: *Phormidium*-type (Cyanobacteria, freshwater strains) | Sucrose and/or trehalose |  |  | [86] |
| Bacteria: *Coleofasciculus chthonoplastes* (Cyanobacteria, marine strain) | Glycosylglycerol (O- α-D glucopyranosyl- (1→2)-glycerol), glucosylglycerate or proline |  |  | [21] |
| Bacteria: *Aphanothece* (Cyanobacteria, halophilic) | glycine betaine, L glutamate betaine (N-trimethyl-L glutamate), glucosylglycerate or proline; sucrose and trehalose, ectoine |  |  | [20,87] |
| Algae: *Dunaliella parva*; *D. salina*; *D. viridis* (Halophilic) | Glycerol | Increase in PG, increase in GL, a higher ratio of C18 to C16 fatty acids. At high salinity fatty acid chain elongation and increased overall desaturation. High amount of sterols | Hyperosmotic shock leads to rigidification, a hypoosmotic shock to transient fluidization and increased sterol content | [88,89] |
| Algae: *Neochloris oleoabundans* (Freshwater strain) | Proline | No significant changes in fatty acid composition |  | [30] |
| Algae: *Scenedesmus* sp. IITRIND2 (Freshwater strain) | Proline, glycine, betaine | Increase in the MGDG. Increase in MUFA’s content was recorded under saline conditions | Maintaining the fluidity of cell membrane | [91,92] |
| Algae: *Monoraphidium dybowskii* LB50 (Freshwater strain) | Galacturonic acid, trehalose, N-acetyl-β-alanine and organic acid | Increase in PL, reduce in GL. Increase the saturation of FAs and extend the long-chain FAs. | Increased fluidity and permeability at optimal salinities | This study |
| Protists: *Halocafeteria seosinensis* (Stramenopiles) | Hydroxyectoine and myo-inositol (synthesis and uptake; based on genomics and gene expression studies) | Increase PI, lowered PE; increase shorter fatty acid chains; repressed fatty acid desaturases. Lower amounts of sterols | Increased fluidity | [93] |
| Fungi: *Hortaea werneckii* (Halotolerance) | Glycerol, Erythritol, arabitol and mannitol; mycosporine glutaminol glucoside (only at lower salinities) | Increase in fatty acid unsaturation | Lowered fluidity at <5% and >15%; highest fluidity at optimal salinities | [5,94] |
| Fungi: *Saccharomyces cerevisiae* (Salt sensitive) | Glycerol | Almost unchanged total sterol content; significantly higher sterol-to-phospholipid ratio than in halophilic fungi | Increased fluidity at salinities that exceed the optimal range | [95] |
| Plant: *Arabidopsis thaliana* (Salt-tolerant) | Proline, polyamine, mannitol | Increased the content and double-bond index of unsaturated fatty acids |  | [9] |
| Plant: *Zea mays* | Trehalose | Increased PI and PG, decreased PE; increased C18:3 and C25:5 |  | [96] |
| Plant: Rice (*Oryza sativa*) | Polyamine, proline, glucose, fructose, trehalose | Increased phosphatidic acid and phosphatidylinositol bisphosphate, decreased CL | Permeability increased | [97] |
| Plant: *Phragmites australis* | Proline, sucrose, fructose |  |  | [98] |

Phosphatidylcholine (PC), phosphatidylethanolamine (PE), phosphatidylglycerol (PG), phosphatidylinositol (PI), phosphatidylserine (PS), Cardiolipin (CL). Glycolipids (GL), cyclopropane fatty acids (CFA), monogalactosyldiacylglycerol (MGDG), monounsaturated fatty acids (MUFA), fatty acid (FA).
